# Polyhydroxyalkanoate recovery from newly screened *Bacillus* sp. LPPI-18 using various methods of extraction from Loktak Lake sediment sample

**DOI:** 10.1186/s43141-022-00392-7

**Published:** 2022-08-06

**Authors:** Seid Mohammed, Lopamudra Ray

**Affiliations:** 1 Department of Applied Biology, SoANS, Adama Science and Technology University, Oromia, Ethiopia; 2 School of Law, KIIT University, Bhubaneswar, Odisha 751024 India

**Keywords:** Carbon source, Isolate, PHA, Producing and species

## Abstract

**Background:**

Nowadays, the conventional plastic wastes are very challenging to environments and its production cost also creates an economic crisis due to petrochemical-based plastic. In order to solve this problem, the current studies were aimed at screening and characterizing these polyhydroxyalkanoate (PHA)-producing isolates and evaluating the suitability of some carbon source for newly screened PHA-producing isolates.

**Material and methods:**

Some carbon sources such as D-fructose, glucose, molasses, D-ribose and sucrose were evaluated for PHA production. Data were analyzed using SPSS version 20. The 16SrRNA gene sequence of these isolates was performed. These newly isolated taxa were related to *Bacillus* species. It was designated as *Bacillus* sp. LPPI-18 and affiliated *Bacillus cereus* ATCC 14577^T^ (AE01687) (99.10%). *Paenibacillus sp. 172* (AF273740.1) was used as an outgroup.

**Results:**

*Bacillus* sp. LPPI-18 is a gram-positive, rod-shaped, endospore former, and citrate test positive. This isolate showed positive for amylase, catalase, pectinase, and protease test. They produced intracellular PHA granules when this isolate was stained with Sudan Black B (SBB) and Nile blue A (NBA) preliminary and specific staining dyes, respectively. Both temperature and pH used to affect polyhydroxyalkanoates (PHA) productivity. Bacteria are able to reserve PHA in the form of granules during stress conditions. This isolate produces only when supplied with carbon sources. More PHA contents (PCs) were obtained from glucose, molasses, and D-fructose. In this regard, the maximum mean value of PC was obtained from glucose (40.55±0.7%) and the minimum was obtained from D-ribose (12.4±1.4%). Great variations (*P*≤0.05) of PCs were observed among glucose and sucrose, molasses and sucrose, and D-fructose and sucrose carbon sources for PHA productivity (PP) of cell dry weight (CDW) g/L. After extraction, PHA film was produced for this typical isolate using glucose as a sole carbon source. Fourier transform infrared spectrum was performed for this isolate and showed the feature of polyester at 1719.64 to 1721.16 wavelengths for these extracted samples. The peak of fingerprinting (band of carboxylic acid group) at this wavelength is a characteristic feature of polyhydroxybutyrate (PHB) and corresponds to the ester functional group (C=O).

**Conclusion:**

In this study, newly identified *Bacillus* sp. LPPI-18 is found to be producing biodegradable polymers that are used to replace highly pollutant conventional plastic polymers. This isolate is also used to employ certain cost-effective carbon sources for the production of PHA polymers.

**Graphical Abstract:**

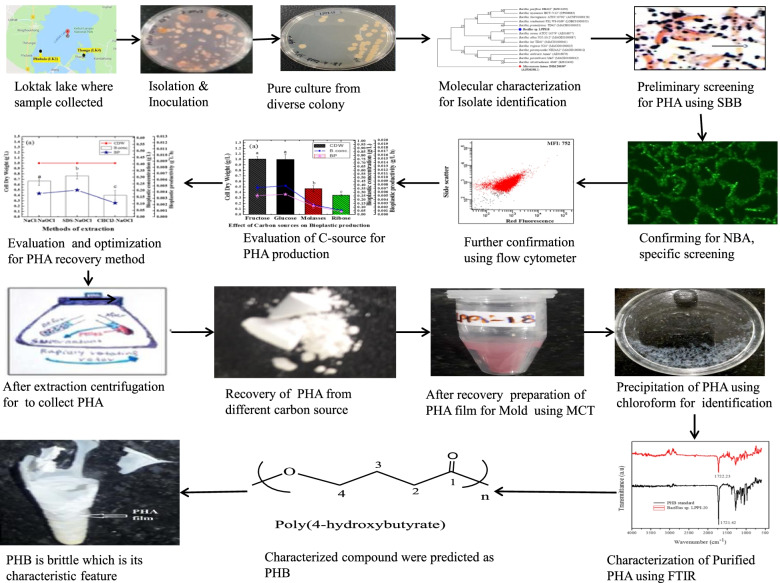

**Supplementary Information:**

The online version contains supplementary material available at 10.1186/s43141-022-00392-7.

## Background

Plastics are moldable organic polymers. They are made up of a wide range of synthetic or semi-synthetic organic solids. Almost all aspects of daily life involve plastics. These plastics play a vital role in transport, for telecommunications, footwear, and packaging materials. They are also used to transport a wide range of food, drink, and other goods [1]. Plastics are also a major raw material for the production of crude oil and natural gas and hence the prices have been boosted since the conventional plastic was derived from petroleum. Recently global demand for an alternative to these non-degradable petroleum-derived plastics has increased tremendously. Over the past few years, bio-based plastics have been developed rapidly owing to rising petroleum prices and due to the possibility that petroleum supplies will be exhausted in the near decades [2].

PHA is commonly known as a polyhydroxyalkanoates. They are biodegradable, biocompatible, and elastomeric. These PHA-producing potential isolates are mostly grown at high temperature. They are able to accumulate compound and hence attracted the world in recent years. PHA is a microbial-based nature product. They are carbon and energy compound stored in some potential strains. All these compounds showed different monomers when they are characterized [3].

PHA are commercially developed and used for marketing purpose. However, the widespread use of these polymers is limited due to high production cost. For instance, the cost of the PHB-co-V resin which is a biodegradable polymer is about $30/kg, which is costlier than conventional plastic ($2/kg) such as polypropylene. Polyhydroxyalkanoate (PHA) readily extracted from respective microorganisms using different chlorinated hydrocarbon. Some methods of extraction are adopted for PHB recovery. These methods are achieved by using chloroform (CHCL_3_), sodium hypochlorite (NaOCI), dispersion of NaOCI and CHCL_3_, and sodium dodecyl sulfate (SDS) (10g/L) [4, 5].

Various groups of PHAs producing bacteria are reported. It was stated that certain *Bacillus* species such as *Bacillus aquimaris*, *B. bataviensis*, *B. flexus*, *B. vallismortis*, *B. vietnamensis*, and *B. megaterium* A12ag which were obtained from saline soil used to produce PHA. It was reported [6] PHA (PHB) contents from inactive and less active strains of *Rhizobium phaseoli*, *R. meliloti*, and *R. trifolii* are able to grow on carbon and nitrogen sources.

Recently, some strategies are developed to produce more PHA polymers from various lignocellulosic wastes. These wastes include cellulose, hemicellulose, and lignin after these wastes have been hydrothermally treated. These wastes are found to be employed as a main carbon source for PHA biosynthesis [7]. Other carbon sources able to produce PHA are glucose, fructose, maltose, and xylose. These carbon sources can be utilized with *Cupriavidus* sp. KKU38 for PHA production and its PHA content is 44.71–73.88% [8]. It was presumed that PHA-producing potential isolates were common in soil, sediment, area of plastic waste, and limited nutrients. It was hypothesized that these isolates were able to use carbon source for PHA polymerization. These PHA extract may vary in terms of its composition and /or type of monomers. Therefore, the present study was aimed at screening PHA-producing isolates, to evaluate suitable and gross PHA-producing carbon source, to extract PHA compound from respective isolates and to characterize the extract of these compound and evaluation of gross PHA-producing carbon sources.

## Methods

### Sample and media preparation

The soil sediment samples were collected from Loktak Lake since diversity of microbial cells is highly expected from soil sediment. The samples were then transported to laboratory for analysis: nutrient agar (NA) (consisting of (all in g/L) peptone, 5; NaCl, 5; yeast extract, 1.5; and agar, 15) were used for seed inoculate preparation.

### Seed inocula preparation

A single Nile blue A-positive isolate with high intensity of PHA granules were selected and aseptically inoculated into 5 ml Nutrient broth (consisting of (in g/L): peptone, 5; NaCl, 5; and yeast extract, 1.5 at pH 7) supplied with glucose (1%) as a carbon source. The cultures were incubated at 37 °C for 24 h.

### Flask fermentation

A 2.5% of seed inocula were taken and transferred to 500 ml conical flasks containing 200 ml sterilized Minimal Davis broth medium (per liter) (1.0 g dextrose, 7.0 g K_2_HPO_4_, 1.0 g (NH_4_)_2_SO_4_, 0.5 g sodium citrate, 2.0 g KH_2_PO_4_, 0.1 g MgSO_4_.7H_2_O, and pH 7.2 ± 0.2 at 25 °C), a PHA production medium. The medium was supplemented with 1% carbon sources (D-fructose, glucose, molasses, D-ribose, and sucrose) (w/v) and incubated at the different temperature on a rotary shaker at 150 rpm for 72 h.

### Staining cells for NBA and SBB

Preliminary staining for PHA-producing isolates was characterized using Sudan Black B, lipophilic dyes [9, 10]. Secondary screening for PHA-producing bacteria using NBA dye was used to confirm for SBB staining [4, 11].

### Characterization of PHA-producing using flow cytometer

Flow cytometry was performed to examine the binding of the tendency of Nile blue A (NBA) to *Bacillus* sp. LPPI-18 [12] with modification. Briefly, 10 ml of Minimal Davis Broth was inoculated with a colony of *Bacillus* sp. LPPI-18 using a 50-ml tube, and cells were incubated for 24 h at 37 °C and 150 rpm. Afterward, a 24-h-old (200 μl) bacterial suspension collected culture and inoculated with 50 ml of Minimal broth Davis containing NBA (0.5 μg/ml final concentration) in a 200-ml Erlenmeyer flask. A similar procedure was conducted for cells without the NBA. Then, 2 ml of culture was taken from a flask containing unstained or stained cells and was centrifuged at 6000 rpm for 10 min at 27 °C. The pellet was washed 3 times with sodium phosphate buffer saline (PBS) (pH = 8). Finally, the optical density at 578 nm (OD_578nm_) was adjusted to 0.2 by adding appropriate PBS, and the samples were kept on ice for flow cytometric analysis. Cells were acquired in BD FACS Canto II using the following instrument settings. Nile blue A: Channel APC = Emission wavelength = 680, log, excitation 633 nm, detection 660/20 nm, threshold 700 FSC and 200 SSC. For each sample, 10,000 cells were analyzed using FACS Diva software.

### Molecular characterization

DNA was extracted. Its concentration was quantified by Nanodrop methods (Biotech Instruments, USA), and PCR was performed for 16srRNA amplification using 16SF-AGAGTT TGATCCTGGCTCAG and reverse primers (16SR-TACGGTTACCTTGTTACGACTT). Gel electrophoresis was run. Bacterial molecular characterizations for the 16S rRNA gene sequence were performed. Following Quick-DNA™Fungal/Bacterial Miniprep Kit (Zymo Research Corporation D6005), the 16SrRNA gene sequences similar to newly isolated bacterial species were also collected from GenBank database using EzTaxon server (https://www.ezbiocloud.net/identify). The *Bacillus* sp. LPPI-18 16S rRNA sequence was submitted into GenBank, and the accession number (ON678114) was obtained from this center. Finally, phylogenetic tree was constructed using MEGA version 7.0.21 software package for identification of these Nile blue A-positive isolate using certain closely related isolates; these are collected from the EzTaxon server which is available at https://eztaxon-e.ezbiocloud.net/tools.

### Biochemical test and enzyme assay

Biochemical test such as gram staining, endospore, methyl red, citrated, and triple sugar agar test was performed. Enzyme activities were also performed for catalase test, amylase tests [13], pectinase test [14], and protease test [15] with little modification.

### Effect of temperature and pH against PHA productions

A Minimal Davis broth was prepared and sterilized at 121 °C for 15 min. After sterilization, 100 ml of broth medium was poured into 250 ml conical flask and supplemented with 1% glucose (w/v) as a carbon source. The bacterial isolates (3%) (v/v) were inoculated into broth medium. The culture was incubated at different temperature (25, 30, 35, and 37 °C) and pH (7, 8, 9, 10, and 11) on a rotary shaker at 150 rpm for 72h. Finally, the pellet was collected by centrifugation (6000 rpm, 15 min at 25 °C), extracted, and quantified.

#### Quantification of PHA contents

Polymer content (%) and productivity (g/L/h) were calculated [16–18]. PHA content (%) = Polymer concentration (g/L) / cell dry weight (g/L) × 100. PHA productivity (g/L/h) = polymer concentration (g/L) / fermentation time (h)

### PHA recovery and it is a purification

#### Recovery of PHA polymers by SDS pretreatment

PHA producers were cultured and biomass was collected by centrifugation (8400 rpm, 10 min at 4 °C). The biomass was dried to constant weight overnight at 50–60 °C. The dried biomass (pellet) was pretreated with an SDS solution (3%) at 55 °C for 15 min. It was centrifuged (8400 rpm, 10 min at 25 °C). The pellet was washed with dH_2_O and centrifuged (8400 rpm, 5 min). The pellet was treated with acetone (100%) for 2 h. It was then air centrifuged and dried at 50 °C to a constant weight. Finally, the PHA contents were recovered [19].

#### Recovery of PHA polymers by NaCl treatment

It was conducted according to [20] with modification. PHA-producing potential isolates were cultured and biomass was collected by centrifugation (8400 rpm, 10 min at 4 °C). The biomass was dried to constant weight overnight at 50–60 °C. The dried biomass (pellet) was pretreated with 6.25M NaCl solution (w/v) at 37 °C for 1 h for PHA recovery. It was centrifuged (8400 rpm, 10 min at 25 °C). Bacterial pellets were washed three times by dH_2_O and centrifuged (8400 rpm, 5 min). The pellet was treated with acetone (100%) for 2 h. It was then air dried. Finally, the PCs were recovered.

#### PHB recovery by using the dispersion of sodium hypochlorite and chloroform

It was performed following the methods of [5]. It was also used for analytical purpose. A 1-g portion of cell powder was treated with a dispersion containing an equal volume of chloroform and a diluted sodium hypochlorite solution (4%) (1:1) (v/v). After the cell powder was treated at 37 °C for 1 h, the mixture was centrifuged (8400 rpm, 20 min, 25 °C) for 10 min, which resulted in three separate phases. The upper phase was a hypochlorite solution, the middle phase contained non-PHB cell material and undisrupted cells, and the bottom phase was chloroform containing PHB. The upper phase was removed first with a pipette, and the middle phase was separated by filtration from the chloroform phase using Whatman filter paper. PHB was recovered from the chloroform phase by non-solvent precipitation (hexane (100%) (v/v). Finally, the chloroform and hexane were removed by evaporation using the oven at 50 °C; the PC contents were determined [18].

### Evaluation of Carbon source for PHA production

#### Effect of carbon sources on PHA production

PHA accumulating isolates were grown in 250-ml conical flasks containing 120 ml sterilized Minimal Davis Broth with different carbon sources (i.e., fructose, glucose, glycerol, molasses, D-ribose, and sucrose) with 1% concentrations. The flasks were incubated at 37 °C on a rotary shaker (150 rpm) for 72 h. After incubation, PHA yields by the isolates were quantified [18].

### Preparation of a PHA film

PHA film was conducted [21, 22] with modification. Totally 40–50 mg of PHA was dissolved in 1 ml of chloroform in microcentrifuge tube. The solution was kept in the oven at 50 °C until complete evaporation of chloroform. Finally, the evaporation of chloroform resulted in the formation of PHA films in the microcentrifuge tube.

### Characterization of PHA

The PHA was dissolved in chloroform and filtered using Whatman filter paper to a new Eppendrof tube. The chloroform was evaporated and dried at 60 °C to a form solid crystal. The crystal of PHA was placed on a polytetrafluoroethylene plate to form a thin layer film which was used to the transmission IR spectra measurement. The transmission IR spectra were measured on a Fourier transform infrared (FTIR) FTIR-8300 spectrophotometer (Shimadzu Co., Japan) scanning from 400 to 4000 cm^−1^. The data was collected and processed using the original software (Wang et al. 2011).

## Results

**Fig. 1:**
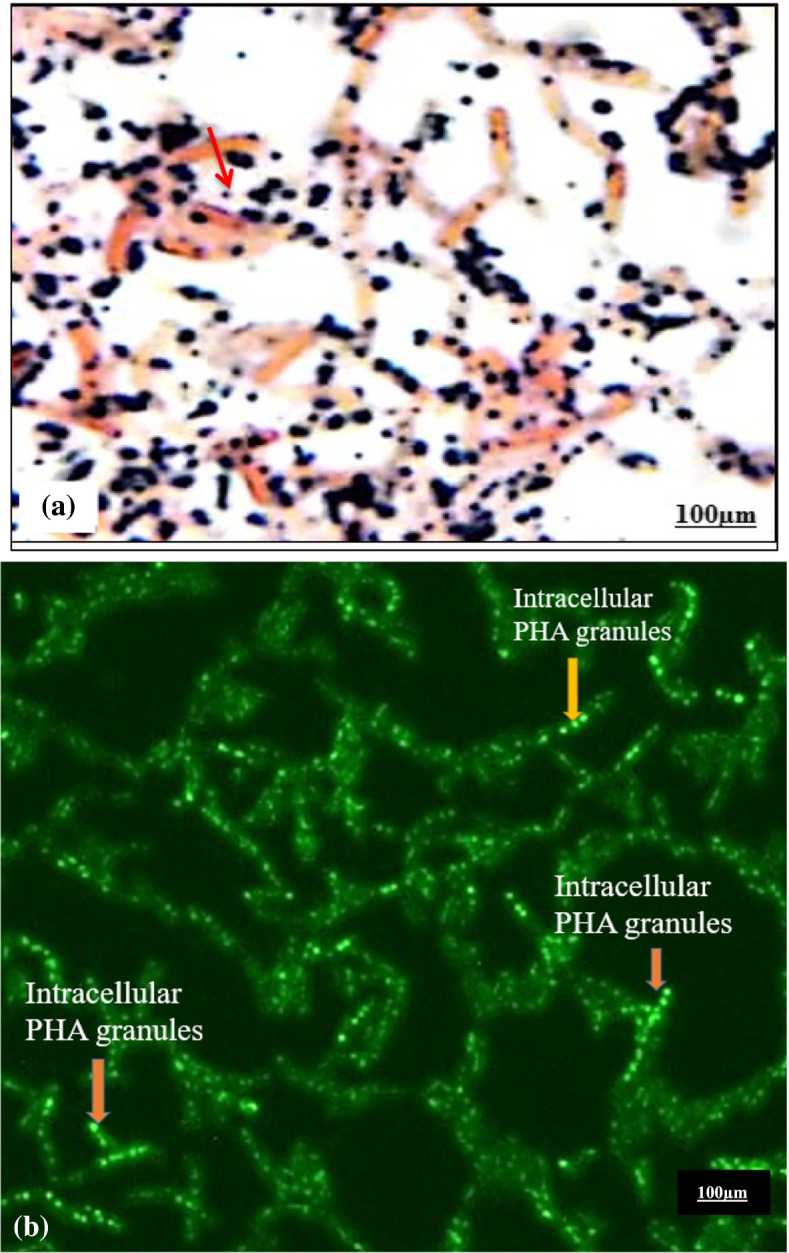
Sudan Black B (SBB) and Nile blue A staining for PHA-producing *Bacillus* species slide staining. **a ***Bacillus* sp. LPPI-18 is a PHA-positive isolate with a better indication of SBB positivity for slide staining. **b ***Bacillus* sp. LPPI-18 is an NBA-positive endospore former obtained from Loktak Lake sediments and stained with 1% NBA dye

A sample collected from Loktak Lake sediment harbored PHA-producing isolate after it had been screened using SBB and NBA, preliminary and selective staining methods, respectively. This isolate was designated as LPPI-18. From the point view of the gram staining, the relative sequence of its 16srRNA, and phylogenetic tree outcome, this bacterial isolate was found to be belonging to *Bacillus* species. As indicated in Fig. 1, the isolate was found to be shown positive for PHA granules when it had been stained with SBB and NBA dyes. This isolate was recognized to be a PHA polymer producer when extraction had been performed using different digesting chemicals and reagents. Its ability for PHA production was also checked against temperature, carbon source, and methods of recovery for dry cell weight.

### Staining cells for NBA and SBB

**Fig. 2:**
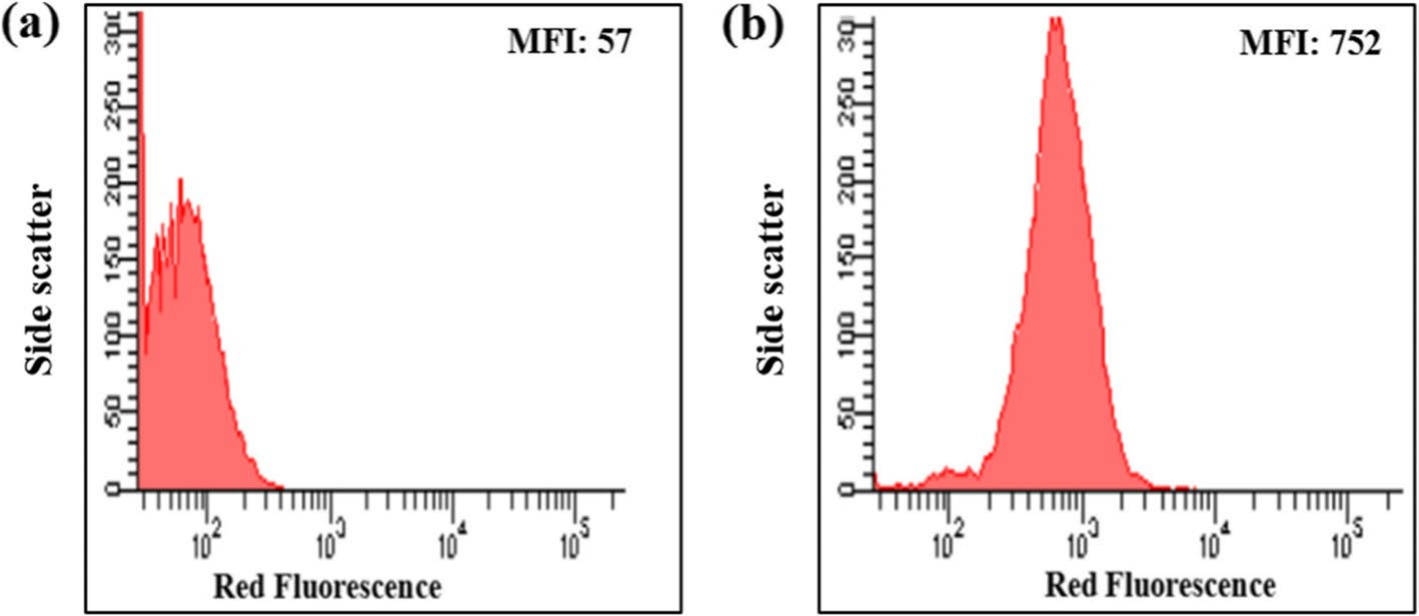
Histogram plots showing binding of NBA to *Bacillus* sp. LPPI-18 obtained from Loktak Lake sediment. **a** Unstained culture of *Bacillus* sp. LPPI-18 with 57 MFI and **b** stained culture of *Bacillus* sp. LPPI-18 with 752 MFI in the presence of NBA at final concentration of 0.5 μg/l

PHA-producing bacterial isolates were preliminary screened using SBB from their natural ecosystems. Upon staining cells by using SBB, the granules are clearly visible in the form of an ordered pattern (Fig. 2). Sometimes, the intracellular granules of this isolate were showed outside cell membrane after stained when observed under the microscope (Fig. 1a) suggesting that during smear preparation the cell wall structure of bacteria may burst and the intracellular granules leaked out of bacterial cells.

SBB is non-specific dye to screen PHA-producing isolates [11]. In this study, it was shown that there was a good indication of SBB positivity when the isolate was collected from sediment and then stained with SBB dyes. In this respect, *Bacillus* sp. LPPI-18 has shown strong SBB positivity after this PHA-producing isolate had been stained with SBB dye (Fig. 1).

In this study, it was found that NBA used to confirm the collection and presence of PHA granules. As indicated in Fig. 1b, this isolate contains intracellular PHA granules when stained with 1% of NBA solution and dried at 55 °C for 15 min in a glass slide Coplin staining jar for 10 min. The slides were then destained with a 0.8% acetic acid. Finally, the granules were observed as green fluorescence (Fig. 1b). The granules were clearly visible when it was observed under fluorescence microscope using oil immersion with a ×100 magnification power (Fig. 1b) in an ordered pattern.

This study shows that more granules were evident for *Bacillus* sp. LPPI-18 isolate and fluorescence intensity was also high at 48 h (Fig. 1). This may lead to high production of PHA contents per gram of CDW. In Fig. 1b, the exact location or position of granules for PHA producers was observed when this isolate was stained NBA, specific dye for screening PHA-producing isolate.

### Characterization of PHA production using a flow cytometer

Flow cytometric analysis showed that *Bacillus* sp. LPPI-18 showed binding to the NBA and the extent of binding was expressed in terms of mean intensity of fluorescence (MFI). When the NBA binds to specific granules in bacteria, the PHA-producing isolate emit fluorescence and the mean values of intensity increased in multiple folds. In this study, the MFI for unstained cells of *Bacillus* sp. LPPI-18 (red fluorescence) was 57 (Fig. 2a, b). On the other hand, the MFI for NBA-stained bacteria was 752 for red fluorescence. There were 13.1-fold increases in MFI for a stained population of *Bacillus* sp. LPPI-18 as compared to cells without NBA indicating a greater affinity of the dye to *Bacillus* sp. LPPI-18 (Fig. 2a, b).

**Fig. 3:**
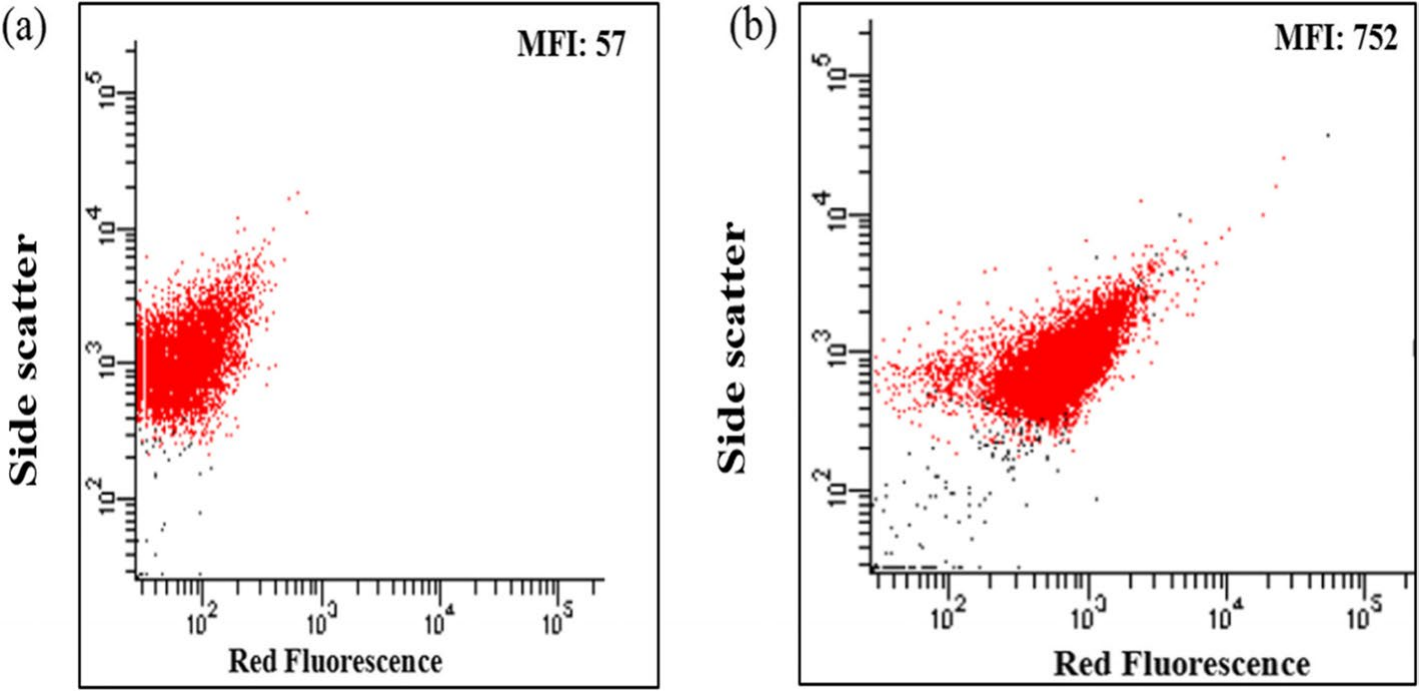
Flow cytometer analysis of *Bacillus* sp. LPPI-18 stained with Nile blue A (0.5μg/L final concentration of NBA dye in Minimal Broth Davis broth). **a** Unstained culture of Bacillus sp. LPPI-18 for side scatter. **b** Stained culture of *Bacillus* sp. LPPI-18 for side scatter. The stained population increases from 57 to 752 indicating that 13.19 multiple fold increased after had been stained with NB

Flow cytometer analysis of unstained and stained with Nile blue A for *Bacillus* sp. LPPI-18 was depicted in Fig. 3. The mean value of relative fluorescence intensity of the histogram plot of *Bacillus* sp. LPPI-18 has been detected. When the cells have been stained with NBA, the fluorescence intensity increases for both isolates with a multiple fold change which is similar work conducted with [12].

### Phylogenetic tree

The 16SrRNA gene sequence similarity with other NCBI database and EzTaxon-e searching tool [23] showed that this isolate was belonging to *Bacillus* species. It was designated as *Bacillus* sp. LPPI-18 (ON678114). The 16SrRNA gene sequence of this isolates mostly identified and affiliated with *Bacillus cereus* ATCC 14579^T^ (AE016877) (99.01%) strains. However, whole genome sequenced *Bacillus cereus* ATCC 14579^T^ (AE016877) (100%) strains do not have a PHA-producing genes such as *phaC*, *phaE*, and *phaR.* It was also biochemically (Table 1) confirmed that the recent isolate is different from *Bacillus cereus* ATCC 14579^T^ (AE016877) type strains which indicates that it could be another strain type.

**Table 1:** Biochemical test and enzyme assay

No	Type of assay	*Bacillus* sp. LPPI-18	*Bacillus cereus* ATCC 14579^T^ [24–26]
1	Isolate source	Soil sediment	Dairy
2	Morphology	Short rod	Rod shaped
3	Temperature for growth	25–37 °C	10–45 °C
4	Fresh plate culture	No smell	Mousy smell
5	2M NaCl	–	NP
6	Colony color	White	White to cream and sometimes pinkish brown
7	1.5 M NaCl	–	NP
8	pH range	7–11	7
9	Gram staining	+	+
10	Endospore	+	+
11	Citrate test	+	+
12	TSI test (H_2_S production)	+	–
13	Bile salt	+	+
14	Methyl red test	+	+
15	Enzyme		
15.1	Amylase test	–	
15.2	Catalase test	+	+
15.3	Pectinase test	+	+
15.5	Protease test	+	+
16	Gelatinase	–	+
17	D-fructose	+	–
18	D-glucose	+	–
19	D-ribose	+	+
20	Glycerol	–	+
21	PHA production on glycerol	–	NP
22	PHA production on crude oil waste	–	NP

*MA* MacConkey agar, *LPPI* Loktak Lake phumula PHA isolates

**Fig. 4:**
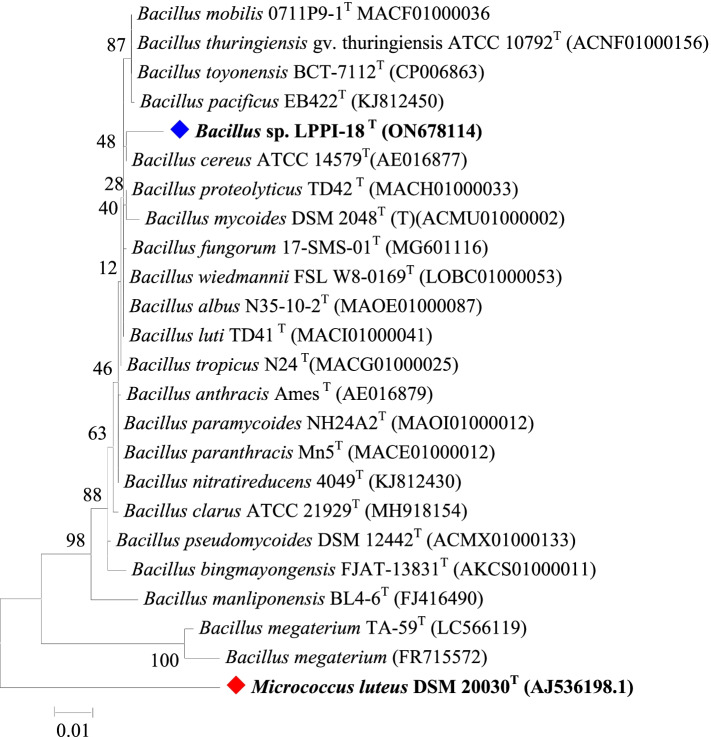
Evolutionary relationships of taxa and phylogenetic tree for 16S rRNA gene sequences of PHA-producing *Bacillus* sp. LPPI-18 (ON678114). The evolutionary history was inferred by using the maximum likelihood method based on the Kimura 2-parameter model [27]. The tree with the highest log likelihood is shown. The percentage of trees in which the associated taxa clustered together is shown next to the branches. Initial tree(s) for the heuristic search were obtained automatically by applying neighbor-joining and BioNJ algorithms to a matrix of pairwise distances estimated using the maximum composite likelihood (MCL) approach, and then selecting the topology with superior log likelihood value. The tree is drawn to scale, with branch lengths measured in the number of substitutions per site. The analysis involved 24 nucleotide sequences. Selected codon positions were 1st+2nd+3rd+Noncoding. All positions with less than 95% site coverage were eliminated. That is, fewer than 5% alignment gaps, missing data, and ambiguous bases were allowed at any position. There were a total of 1384 positions in the final dataset. Evolutionary analyses were conducted in MEGA 7.0.21 [28]. The replication of confidence values was 1000 bootstrap (percentage of 1000 replication). Bar, 0.01 substitutions per nucleotide position. *Micrococcus luteus* DSM 20030^T^ (AJ536198.1) was designated and used as outgroup in the analyses; other related sequences were obtained from EzTaxon-e server

The phylogenetic tree with the maximum likelihood method showed that *Bacillus* sp. LPPI-18 formed clad with *Bacillus cereus* ATCC 14579^T^ (AE016877) (99.01%) strains with a bootstrap value of 52 scores. A comparative 16srRNA gene sequencing analyses indicated that *Bacillus* sp. LPPI-18 is also closely affiliated with *Bacillus wiedmannii* FSL W8-0169^T^ LOBC01000053 (98.94%) and *Bacillus paramycoides* NH24A2^T^ MAOI01000012 (98.94%) (Fig. 4). In this study, the GC contents (%) for the recent isolate and other closely related isolates were also predicted and shown variously. For instance, the CG contained found to be 53.10% for *Bacillus* sp. LPPI-18 (ON678114) whereas the CG contains *Bacillus cereus* ATCC 14579^T^ (AE016877) found to be 53.53%. This indicates that the recent isolate shown to be various when it was compared to the closest strain, *Bacillus cereus* ATCC 14579^T^ (AE016877). Furthermore, the recent isolate and other closely related strains are also shown to be various (Additional File 1: Table S1).

### Biochemical tests and enzyme assay

Few biochemical tests were performed for these SBB- and NBA-positive isolates (Table 1). It was a gram-positive and rod shaped in morphology. All Nile blue A-positive isolate obtained from sediment of soil sample showed proteolytic activities after incubation for 48 h at 37 °C (Table 1). They are able to show protease-positive isolates when growing on nutrient agar that is supplemented with 1% skim milk.

### Effect of pH and temperature against PHA productions

**Fig. 5:**
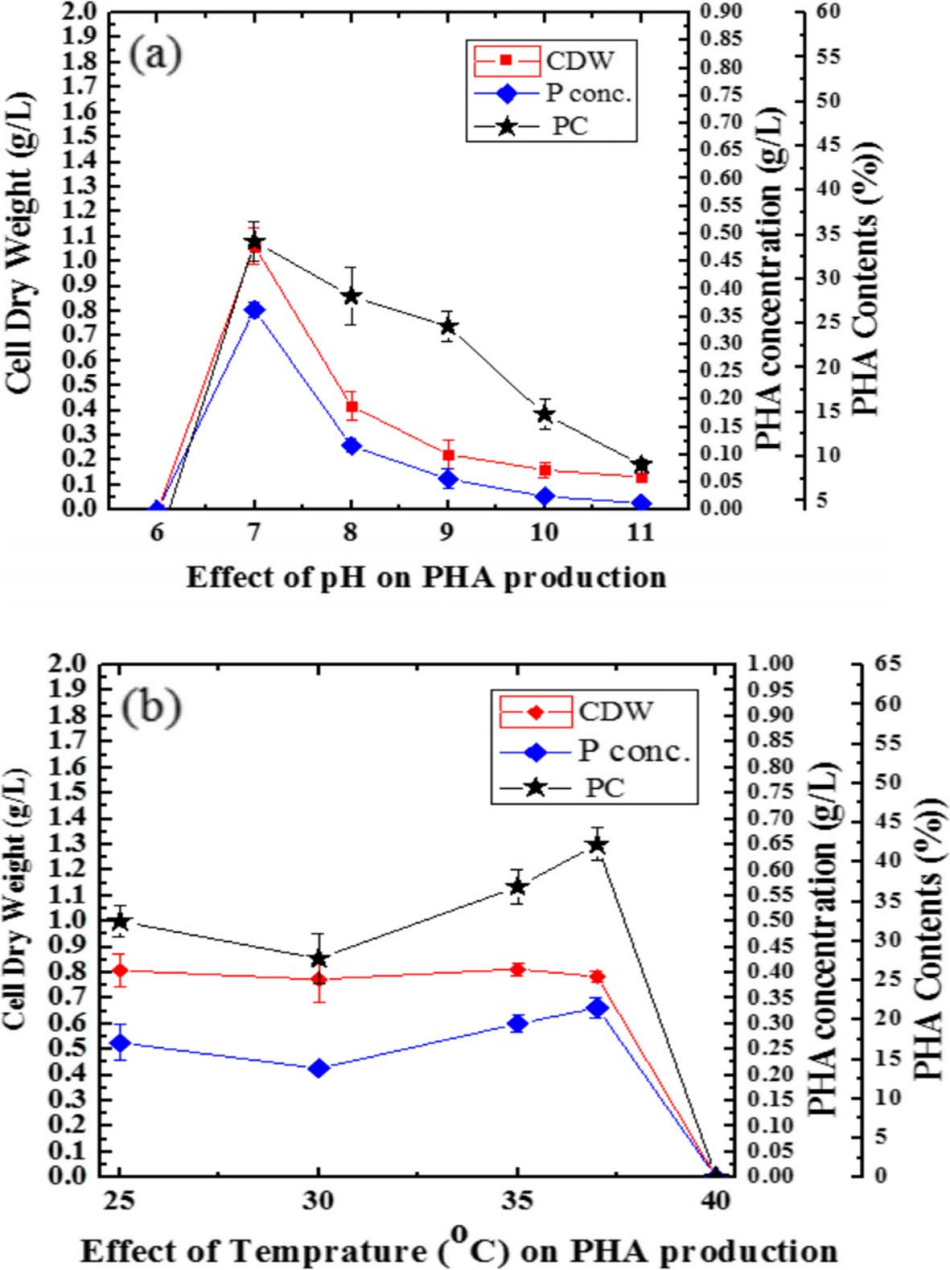
**a** PHA contents (PCs) for *Bacillus* sp. LPPI-18 over a range of 7–11 pH value. **b** PHA production at a different temperature when 1% glucose was supplemented as a carbon source. Data represented the mean ±SD of three replicate (*n*=3) of PHA contents (PCs)

Physical parameters such as temperature and pH used affect microbial growth and hence natural products. In the present study, over the range of 9.08±0.6 to 34.25±2.26%, PCs were obtained between 6 and 11 pH (Fig. 5a). Different PCs were recorded at different pH values. In this study, the was minimum (9.08±0.62%) detected at pH 11 and maximum (34.25±2.26%) PC was recorded for *Bacillus* sp. LPPI-18 at 7 pH (37 °C) (Fig. 5).

The present study has shown that *Bacillus* sp. LPPI-18 was able to grow at pH 11. However, they produced fewer amounts of PCs (Fig. 5a) at 11 pH. Roughly 0.132±0.002 g/L to 1.061±0.074g/L cell dry weight (CDW) was obtained from this isolate with 0.012±.001% to 0.362±0.014% P. conc. (g/L) (Fig. 5a) when the glucose was supplied as a sole carbon source. A considerable amount of PCs was obtained for this isolate at 35 (36.89±2.14%) and 37 °C (42.23±3.49%) (Fig. 5b) for *Bacillus* sp. LPPI-18 with 0.3±.016 to 0.36±0.01 P. conc. of CDW (g/L). However, at 25 and 30 °C, less amounts of PCs were obtained for these PHA accumulators (Fig. 4) with 0.77±0.09 to 0.81±0.06 CDW (g/L) (Fig. 5b).

### PHA recovery and its purification

In this study, it was revealed that bacterial cells contain PHA granules. It is either single or many in number (Fig. 1). The existence of these granules is enhanced by extraction processes. In this study, the extractions were conducted by SDS-NaOCl, CHCl_3_-NaOCl, and NaCl-NaOCl methods for both recovery and purity of polymers from respective bacterial cells.

#### PHA recovery by using dispersion of chloroform and sodium hypochlorite

**Fig. 6:**
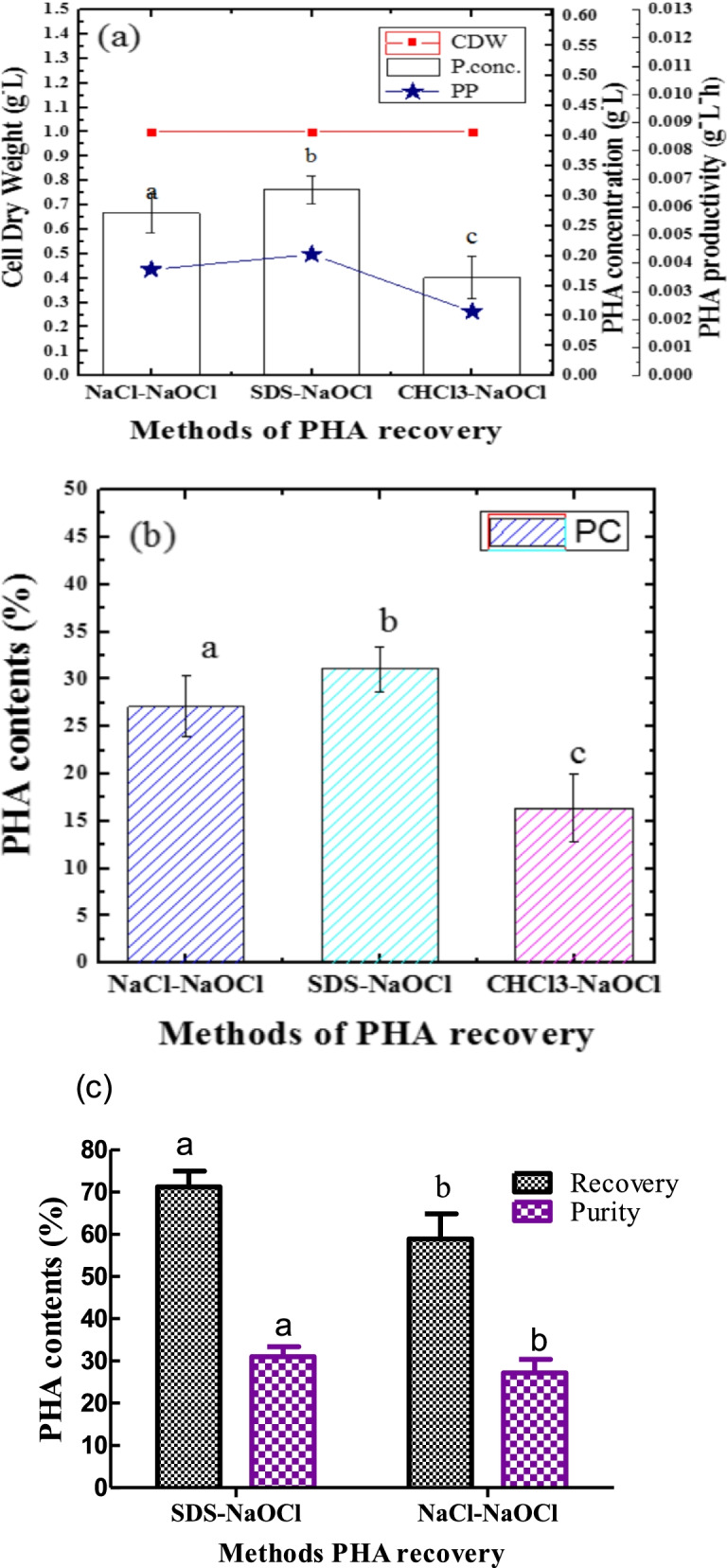
**a** Effect of SDS-NaOCl, CHCl_3_-NaOCl, and NaCl-NaOCl solution on CDW, P. conc., and PP. **b** Effect SDS-NaOCl, CHCL_3_-NaOCl, and NaCl-NaOCl solution on PC from 1 g CDW of *Bacillus* sp. LPPI-18 is that isolated from sediment. **c** Recovery of PCs using SDS (3%) and NaOCl solution and recovery level of PCs with 6.25 M NaCl and NaOCl solution. Data represented the mean ±SD of three replicates (*n*=3). According to one-way ANOVA, the CDW (**a**), P. conc. (**b**), and PC (**c**) have significant variation (*P*<0.05) for methods of extraction having the different latter on their respective bars

In this study, it was indicated that CHCl_3_-NaOCl is a method of PHA recovery. It was used for analytical purposes. A 16±3.59% of PC with 0.16±0.04 g/l of PHA concentration (P. conc.) was obtained from 1 g of CDW (g/l) (Fig. 6a,b) using glucose as a sole carbon source.

#### Recovery of PHA polymers by SDS pretreatment

PHA content (PC) which is PHA concentration (g/L) / DCW X100% was produced from a 60-h-old *Bacillus* sp. LPPI-18 culture using batch fermentation methods. It was recovered using SDS-NaClO treatment. Figure 6 shows that less amounts of PC (31.05±2.33%) have been obtained with 71.20±3.26% level of recovery when SDS-NaOCl methods of extraction were used. Figure 6 a and b show that less amounts of P. conc. and PC which are lower than PC obtained from CHCl_3_-NaOCl probably result in less molecular weight of PHA polymer.

#### NaCl-NaOCl treatment for PHA recovery and purification

Sodium chloride was also used to recover PHA polymers from bacterial biomass. The PHA content (PC) was less when compared to CHCl_3_-NaOCl and SDS-NaOCl methods of extractions. A 27.13±3.26% purity of PC with a 68.54±3.08% level of PHA polymer recovery was obtained from *Bacillus* sp. LPPI-18 under suitable condition (Fig. 6) at 6.25 M NaCl concentration. Figure 6c shows 1 g of CDW is used to give fewer amounts of PHA contents under the suitable environmental condition when glucose is used as a sole carbon source.

### Evaluation of carbon source for PHA production

A newly isolated *Bacillus* sp. LPPI-18 has been found to able to accumulate PHA using different carbon sources. This isolate grew on fructose, glucose, molasses, D-ribose, glycerol, and sucrose at a concentration of 10 g/l. However, the obtained yields were variously based on the type of carbon source used under the same environmental condition.

The PCs (%) for PHA-producing isolates were analyzed using one-way ANOVA (paired samples test). Significant variations (*P*≤0.05) were detected among PHA contents (%) (D-fructose (1%) & glucose (1%)) (D-fructose (1%) & sucrose (1%)), glucose (1%) and molasses (1%), glucose (1%) and D-ribose (%) (glucose (1%) & sucrose (1%)), and molasses (1%) and PHA contents (%) for sucrose (1%) at 95% confident interval. The PHA accumulating ability of this isolate was also determined by these carbon sources.

#### PHA productivity against fructose (1%) as a carbon source

**Fig. 7:**
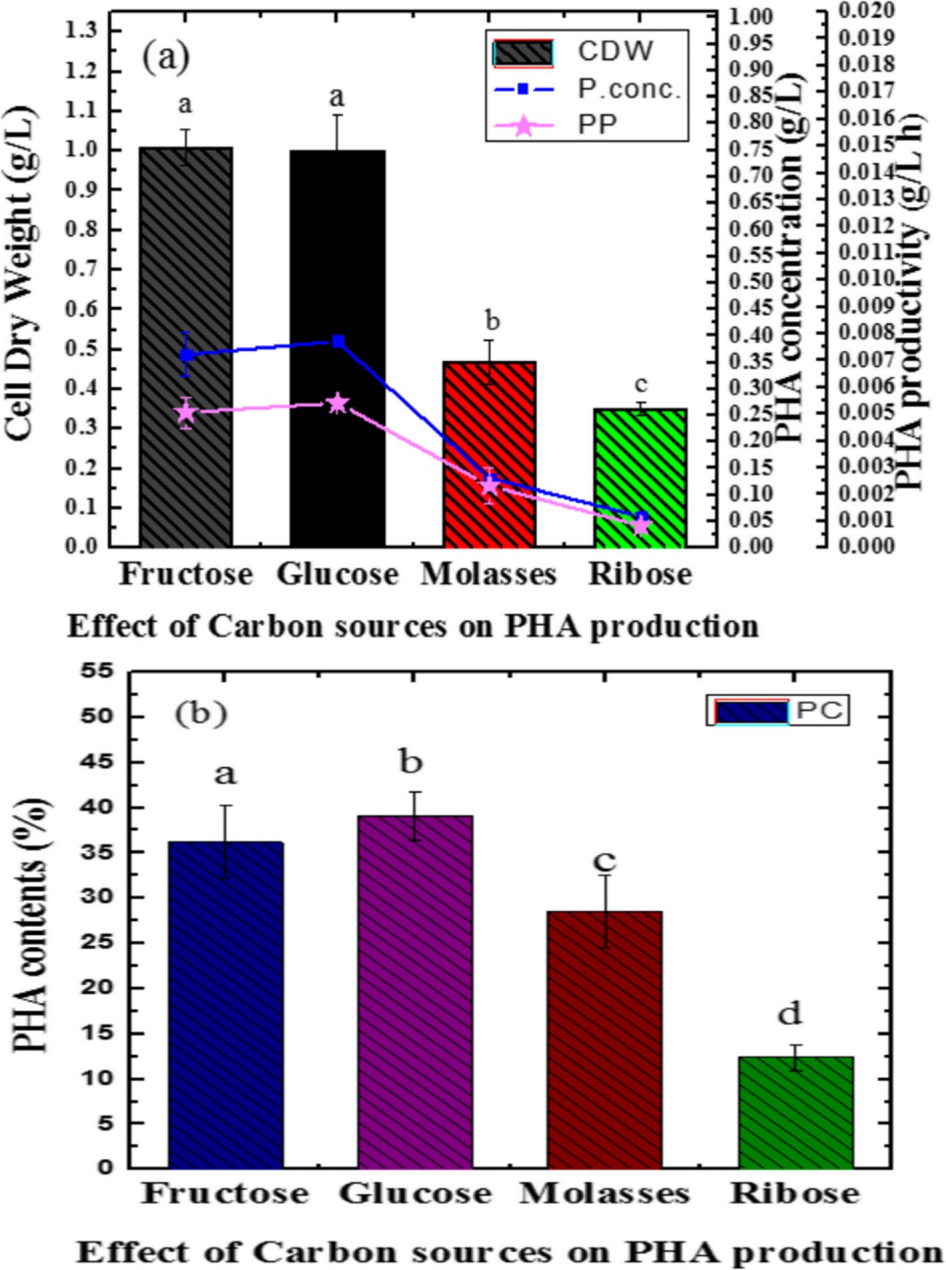
**a** Effect of different carbon sources on CDW, P. conc., and PP for newly isolated *Bacillus* sp. LPPI-18 that are obtained from soil sediment of Loktak Lake. **b** PCs for 1% fructose, glucose, molasses, and ribose against *Bacillus* sp LPPI-18 at 37 °C. Data represented the mean ±SD of three replicates (*n*=3). One-way ANOVA indicates that the PC has no significant variation (*P*≥0.05) for isolate having the same latter on their respective bars

Bacteria are able to grow on simple sugar such as fructose. In the study, a new isolated *Bacillus* sp. LPPI-18 is able to use fructose and produce PHA polymers. In Fig. 7a,b, 36.19±3.97% has been obtained with 0.36±0.041 g/l of P. conc. of CDW.

#### PHA productivity against glucose (1%) as a carbon source

Glucose was used as a carbon source for PHA accumulation. These isolates are able to produce PCs when the medium was supplemented with glucose (1%) as a carbon source in the deficiency of other nutrients such as phosphate, nitrate, and sulfate. In this study, glucose was found to give 37.00±1.67% PC when MDA had been utilized as a suitable medium for *Bacillus* sp. LPPI-18 growth (Fig. 7). When compared to other carbon sources (Fig. 7), glucose is by far the best carbon source for *Bacillus* sp.

#### PHA productivity against molasses (1%) as a carbon source

The most challenging for PHA production is the cost of the substrate which is as a carbon source. As an alternative, wastes like molasses play a vital role for PHA production. In this respect, sugar cane molasses is able to produce PCs. However, the production was based on potent isolates. In this study, the maximum PC accumulation occurred from 60 to 72 h for these isolates. Molasses was found to be used as a carbon source for bacterial growth and PC accumulation. In Fig. 7a,b, a PC obtained from molasses is 28.53±3.96% with 0.48± 0.055 g/l CDW from *Bacillus* sp. LPPI-18. The study was revealed that molasses are a cheap carbon source that are able to give PHA yield at low concentration of molasses (10 g/L) at 37 °C (pH 7).

#### PHA productivity against D-ribose (1%) as a carbon source

A 10 g/L of D-ribose was supplemented separately as a sole carbon source in 1000 ml of minimal Davis broth medium and incubated at 37 °C for 48–60 h. After biomass collection and having undergone extraction, the mean value of PHA contents was recorded for *Bacillus* sp. LPPI-18 (12.40±1.41, 0.139g/L of CDW). One-way ANOVA (paired samples test) has shown that D-ribose has significant variation than among fructose, glucose, and molasses (*P*≤0.05). The PC obtained from these PHA accumulators were varied (*P*≤0.000) when these isolates were grown on D-ribose substrate as a carbon source. D-ribose is a 5-carbon sugar that enables PHA-producing bacteria to accumulate PHA. However, the production level for PHA is less suggesting that it may be used for other cellular function when compared to fructose, glucose (1%), and molasses with significant variation (*P*≤0.05). Probably, D-ribose may need many enzymatic reactions for catabolisms and reserve carbon compound used to develop PHA granules. For instance, *Bacillus* sp. LPPI-18 produces the highest cell dry biomass (1.13±0.11%) even though very less PC and P conc. were obtained (Fig. 7). It was observed that D-ribose used to give less amounts of PHA yield when it had been supplied as a main carbon source.

#### PHA productivity against sucrose (1%) as a carbon source

All the bacterial species do not have the same ability to utilize sucrose as a carbon source. Some utilized very efficiently, but some are still unable to utilize and produce PHA. In this study, *Bacillus* sp. LPPI-18 are unable to produce PHA polymers when sucrose (%) was used as a main carbon source. Its ability to utilize diverse carbon source may be limited. It has also been observed that sucrose utilization can be used as identification techniques.

PHA contents = PHA concentration (g/L) / DCW × 100%. PHA productivity (g/l/h) = PHA concentration (g/L) /fermentation time (HR) [16]. Biological synthesis (g/g/h) =PHA concentration (g/L) / residual cell (g/L) × fermentation time (h) [29].

### Preparation of a PHA film

**Fig. 8:**
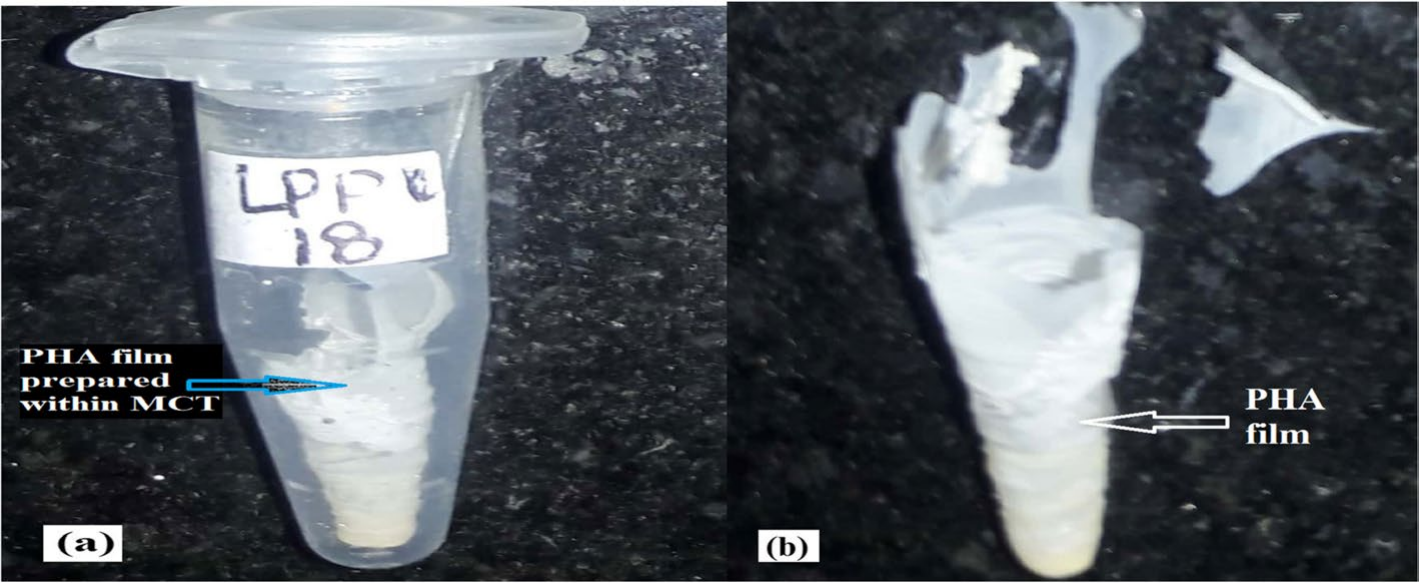
**a** PHA film for *Bacillus* sp. LPPI-18 after treated with SDS-NaClO solution and dissolved within chloroform using microcentrifuge (MCT) as molding material. **b** A partly fractured PHA film after it had dried and was removed from MCT

Bacterial biomass was extracted with an SDS-NaCLO solution and gave white powdered color when this PHA of biomass was dried. When these powdered PHA, which were obtained from different PHA-producing isolates, were kept inside chloroform, they completely dissolved which is characteristic of PHA. However, the produced PHA film was highly brittle. The films of PHA were made as depicted in Fig. 8a. These PHA films are produced from different *Bacillus* species. These films were simple and easily brittle. The type and color of these films are based on the bacterial type and the substrate used for fermentation. In this study, certain carbon sources such as D-fructose, glucose, molasses, D-ribose, and sucrose were used as a carbon source and /or substrates. From glucose, large amounts of films were produced. The more the PHA contents, the larger the film produced. Less film was produced from D-ribose and sucrose. When highly concentrated sodium hypochlorite was used, the PHA granules become digested and no film will be obtained.

### PHA compound characterization using FTIR

The functional group of FTIR for PHA recovery from *Bacillus* sp LPPI-18 isolate is depicted in Fig. 8b along with standard PHB polymer.

**Fig. 9:**
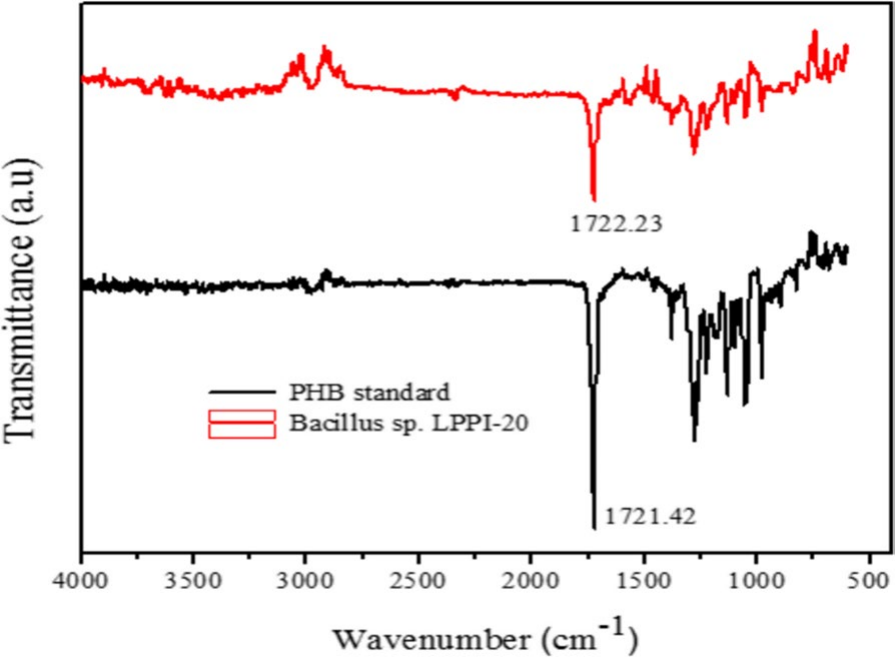
Fourier transforms infrared spectrum of the *Bacillus* sp. BPPI-14 polymer, *Bacillus* sp. LPPI-18, and standard PHB (Sigma). Note: The functional group of FTIR analysis of extracted polymers from *Bacillus* sp. LPPI-18 was closely related to the functional group of standard PHB (Sigma)

The extracted PHA polymers showed an intense absorption peak with C=O bond which is common in carbonyl group at 1722.23 cm^−1^ (Fig. 9) that match with standard PHB (1721.42 cm^−1^) (Fig. 9).

## Discussion

SBB is non-specific dye to screen PHA-producing isolates [11]. In this study, it was shown that there was a good indication of SBB positivity when the isolate was collected from sediment and then stained with SBB dyes. In this respect, *Bacillus* sp. LPPI-18 showed strong SBB positivity after staining with SBB dye.

PHA and non-PHA-producing species of bacteria can be differentiated from each other using various methods [20]. They used to stain viable colony. These methods are quite fast and sensitive and then result in dark blue which is similar to the current study (Steinbuchel and Schlegel, 1991; Bhuwal, *et.al.*, 2013). However, it is time-consuming [11]. When properly stained, distinct black granules with red background due to counterstaining (0.5% safranin (w/v)) were detected. The longer the incubation period, the more granules developed when these isolates were supplied with 1% glucose as a carbon source.

NBA used to discriminate between PHA-producing and non-PHA isolates. Staining dyes are the oldest and fastest technique available to screen for PHA producers from their natural environments. It is the cheapest method of screening potential isolates used for PHA production. The staining dyes used for screening PHA-producing isolate from their natural environments were NBA and Nile Red. In accordance with previous reports, these dyes are the most broadly used dyes for selective staining of PHA granules within bacterial cells [4, 30].

SBB dye has a high sensitivity for PHA screening isolates from their natural habitat (Burdon, 1946). From a diversity of isolates of bacteria, a total of about 42 lipophilic isolates have been screened when stained properly with SBB dye. It is used to discriminate PHA granules with distinct structure. However, SBB positivity was lastly confirmed by using NBA which is a specific dye for PHA granule binding ability [31].

After SBB staining, PHA-positive isolates showed blue-black granules. It was indicated that the PHA-positive isolates have a tendency to store PHA. Following the results of SBB staining methods, the B3-D indicated a considerable amount of PHA granules after heat-fixed and staining had been performed using SBB. The same isolates of bacteria were further checked for PHA production by using NBA dye, a more selective and specific dye for PHA granules. Pure culture of isolates was inoculated and grown on minimal media and incubated at 30 °C for 90 h. Then, the cells were heat-fixed for smearing. Confocal fluorescence microscopic images have shown accumulation of PHA granules within a cell of bacteria [32]. When the cells had been stained with SBB, the granules were observed in the central region and occupying the maximum space within the cell. Moreover, the PHA granules were observed as hollow circles in the typical PHA producers within a microbial cytoplasm when it had been observed under a phase contrast microscope [33].

NBA is also a lipophilic dye most commonly used as a fluorescent probe for staining cells of bacteria that harbor PHA granules. It can be dissolved in water which is polar solvent and has positive charges and hence important for biotechnological application. It was used in the medium with DMSO as an organic solvent. NBA was first spread out to cytoplasm and later into the PHA granules. PHA-positive colonies will fluoresce under ultraviolet irradiation. It was used to discriminate between PHA-producing isolate and PHA-negative strains Therefore, it is the viable colony staining methods [11]. It is a satisfactory stain for PHA granules in bacterial cells. It is also specific and superior to SBB for PHA granule staining. NBA appears to be more selective and specific to stain PHB granules than SBB does. It is not as easily washed from the cell during decolorization procedures [4].

During plate staining methods, the presence of NBA and Nile red dyes have no effect on the growth of cells. This viable colony method of staining is used to apply for both gram-positive (e.g., *Bacillus megaterium* or *Rhodococcus ruber*) and gram-negative bacteria (e.g., *Escherichia coli*, *Pseudomonas putida*, *Azotobacter vinelandii*, and *Ralstonia eutropha*). Both Nile blue A and Nile red have an insignificant role for distinguishing between PHA-negative and PHA-positive isolate such as gram-positive *Bacillus megaterium* or *Rhodococcus ruber*, respectively*.* Normally, it was also used to differentiate between triacylglycerol-negative and triacylglycerol-positive and wax-ester strains of *Acinetobacter calcoaceticus* or *Rhodococcus opacus* [11]. Nile blue A (NBA) is a selective dye used to stain polyhydroxyalkanoic acids producing bacterial isolates. It has been shown that *Escherichia coli* cells are unable to produce polyhydroxyalkanoic acids when they have been stained with NBA and detected with flow cytometry. It is a simple, low-cost, and easy to stain cell using flow cytometry [12].

It was observed that a PHB accumulation ability of *B. cereus* SE-1 and *Bacillus* sp. CS-605 has been determined at 24, 48, and 72 h of interval [34]. The cells of *B. cereus* SE-1 and *Bacillus* sp. CS-605 are detected as bivariate distributions when these isolates were examined with a flow cytometer. It has been observed that more PHA accumulation is detected for isolate *B. cereus* SE-1 than *Bacillus* sp. CS-605 under the same condition after 72 h incubation. For instance, *Bacillus* sp. CS-605 is able to accumulate 5, 18.1, and 33% of PHB at an interval of 24, 48, and 72 h respectively. *B. cereus* SE-1 able to store 3.5, 22.1, and 40% PHB under the same condition [34].

A recent study revealed that the 16SrRNA of *Bacillus* sp. LPPI-18 had shown more affiliation with *Bacillus cereus* ATCC 14579^T^ (AE016877). Our result indicates that this PHA-producing isolate has also shown more sequence similar to that of *Bacillus wiedmannii* FSL W8-0169^T^ LOBC01000053 and *Bacillus paramycoides* NH24A2^T^ MAOI01000012 strains. However, the GC composition and sequence between *Bacillus* sp. LPPI-18 and other closely related have shown variation. This indicates that the recent isolate shows to be various when it was compared to the closest strain, *Bacillus cereus* ATCC 14579^T^ (AE016877). In contrary to this study, it is revealed that [10] the 16SrDNA sequences of E13 and C18 Mat obtained from Polluted Marine Microbial showed a 100% similarity with *Bacillus thuringiensis* DiSz8 sequence which is screened from a polluted sample of soil. The same authors reported that weathered granite have been recognized to be able to use as a source and habitat to harbor for PHA-producing C19 isolates which have shown a sequence similarity (99%) with endolithic *Bacillus megaterium* WN603. When *Paracoccus homiensis* DD-R11 grow on sandy beach sample, they are able to develop PHA granules and their sequence similarity (100%) were affiliated with E33, E45, and E46 strains. The sequence of strain C20R and E63 have been also shown 100% sequence similarity with gram-positive *Staphylococcus cohnii* (GTC) 728 strains. A 100% sequence similarity have been also observed between E4 and *Staphylococcus arlettae* (ATCC) (43957) sequences [10].

Certain isolates are able to produce pectinase enzyme and degrade pectin. In this study, *Bacillus* sp. LPPI-18 produce pectinase and produce a zone of utilization on a minimal salt medium that was supplemented with 1% pectin and incubated at 37 °C for 48 h (pH 7). In an agreement with the present study, it was [35] reported heat tolerance and acidic pectinase-producing *Bacillus* sp. ZJ1407 from soil sample after cultivated for 48 h at 37 °C. *Bacillus* sp. DT7 and *Bacillus* sp. TMF-1 were also other newly isolated bacterial species that are able to produce a thermotolerant pectinase using solid-state fermentation [36, 37].

It was [38] reported that out of 38 yeasts isolates, only *Cyteromyces matritensis* showed a small zone of hydrolysis. The same author further stated that extracellular proteases secreted by yeasts have been investigated for industrial application since they are fast growing and have the ability to grow in diverse substrates. A *Bacillus marmarensis* sp. nov from mushroom compost that was able to produce an alkaliphilic protease enzyme was reported when it had been incubated for 72 h at higher pH than the present study [39]. The present protease enzymes may be neutral protease enzyme. Those authors isolated this novel *Bacillus marmarensis* sp. at lower (30 °C) than the current temperature which is 37 °C. In this study, it shows that this isolate is unable to grow and produce more PCs when increasing the pH value as high as 11. Because of high pH, alkali region will be created and this might suppress these bacterial growths and hence affect PHA intracellular granule formation. In line with this result, it was [40] confirmed that more PHA yield can be obtained at pH12 to reduce the production cost of PHA polymers within a short time span.

The amount of P. conc. obtained from a newly isolated *Bacillus* sp. LPPI-18 is less than PHA contents obtained from *Ps. fluorescens* S48 (55% PHA content) [41] using the same methods of extraction. This could be due to different types of strains, carbon source, and the medium used for PHA productions. The highest amounts of P. conc., PP, and PC are obtained by CHCl_3_-NaOCl dispersion method of extraction when compared to SDS-NaOCl and NaCl-NaOCl methods of extraction. While pretreated with 3% SDS (w/v) and 6.25 M NaCl, PHA polymers may be degraded. This may further result in less amount of molecular weight of PHA polymers which is a similar finding with [5].

In agreement with our estimation, Ramsay et al. [29] obtained less molecular weight (730,000 kDa) and protein (0.7%) percentage with maximum purity of PHB (97%) using 1% SDS-NaClO solution. However, the molecular weight of PHB is 1,200,000 kDa for an untreated sample which is higher than 3% SDS pretreated sample with a less purity of PHB and more percentage of protein (50%) [29]. Sodium hypochlorite solution (4%) was used to degrade non-PHA cellular materials while extracting. The highest amount of PHA was obtained from *R. eutropha* (86%) and recombinant *E. coli* (93%) using NaOCl solution (4%) methods of treatment SDS which is a more PC than the present PC obtained from *Bacillus* sp. LPPI-18. In line with our recent study, a high level of purity (68%) and recovery yield (94%) for PHA polymer were reported from recombinant *C. necator* when a halogen-free method of extraction was used at 30 °C [42].

It was confirmed that there was less purity with the lower recovery of PHA polymer for NaCl-NaOCl methods of extraction when it was compared to 3% SDS and NaOCl-CHl_3_ methods of extraction. It could be due to high osmotic pressure developed against bacterial cell wall by NaCl solution which is 275.106 atm/mol (Π = єMRT) a similar result to [43]. The bacterial cell contents and PHA granules, as a result, were released outside and exposed directly to NaCl solution.

The highest PHA yield (52%) has been recovered from aerobic granules for *Bacillus* sp. using saturated sodium chloride solution (6.84 M) from a 24-h-old culture [20] which has higher yield than our current results for *Bacillus* sp. LPPI-18 (i.e., 27.13±3.26% purity PC). It should be noted that these isolates are able to produce more PC when extracted with SDS-NaOCl and CHCl_3_-NaOCl methods of extraction. *Bacillus* sp. LPPI-18 obtained from Loktak Lake sediment sample gave fewer PCs than those obtained from landfill sites (data not shown). This isolate obtained from sediment may contain a broad range of metabolic diversity for carbon source utilization for PHA polymerization. It was also true that sediment isolates are accessible to various carbon sources in the form of wastes that joined from dry-fill sites.

It was reported that different types of PHA were obtained from varieties of *Pseudomonas putida* strains (Wang et al. 2011). The maximum PHA yield obtained from a strain of *P. putida* KTOY06 (72.4±0.9) [44] is higher than our current results (27.13±3.26% pure PC with a 68.54±3.08% level of PHA recovery). However, still, certain *P. putida* strains are able to produce less PHA polymers under normal condition. This could be a due limitation of a broad range of metabolic diversity (Wang et al. 2011).

Π = єMRT (1 equation) is used to estimate osmotic pressure created against the cell wall of bacteria during extraction time while using 6.25M NaCl solution [43], where *π* is the osmotic pressure (atm), *є* is the van Hoff’s constant for NaCl (1.8), *M* is molarity of NaCl solution, *R* is the gas constant (0.08206 L atm /K mol), and *T* is the room temperature in K (298 K) [20].

Certain *Bacillus* species are also able produce PHA granules. They are also reported to be useful for various applications. The most commonly used *Bacillus* sp. for different applications beside PHA productions were *B. amyloliquefaciens*, *B. brevis*, *B. circulans*, *B. coagulans*, *B. laterosporus*, *B. mycoides*, *B. licheniformis*, *B. macerans*, *B. cereus*, *B. firmus*, *B. subtilis*, *B. sphaericus*, *B. megaterium*, and *B. thuringiensis.* The *Bacillus* species are also more potent than others since they produce homopolymer and copolymer PHAs that increase the diverse nature of the synthesized PHAs [45, 46].

All these carbon sources have not given the same amount of PHA contents. Glucose (1%) was able to produce the maximum PHA contents followed by molasses (1%). The least PC content has been obtained when Minimal Davis broth was supplied with 1% glycerol. These sugars are likely metabolized by this PHA-producing isolate at a very low rate and gradually produce different amounts of PCs. In agreement with the current study, Gouda et al. [47] reported the highest PHB yield from *Bacillus megaterium* using glucose as a sole carbon source. The same authors reported accumulation of PHB in *Bacillus megaterium* using fructose, glucose, and maltose carbon sources. Although the highest PHB yield was obtained from glucose, more cell dry mass was, however, obtained from maltose when it was used as a carbon source for *Bacillus megaterium* [47].

It was stated that [48] between a carbon source used for PHA synthesis, fructose is one of the most important substrate used in polymer (65.37%, 2.18 g/L) production which has more PHA contents than our present results. The same authors further stated that maltose was also able to produce a fewer amount of polymers. Palm olein (PO) (5 g/L) is the other substrate used for PHA production. About 67% PHA copolymers with 3HHx (27 mole %) were obtained from a 5.13 g/L bacterial biomass or cell dry weight (CDW) when *Cupriavidus necator* Re2058/pCB113 pure cultures grow on the PO [49].

It was revealed that these isolates were able to accumulate intracellular PHA granules under suitable condition. Since the fructose is a simple sugar, *Bacillus* sp LPPI-18 easily break down and produce intracellular PHA granules. It was also shown that isolates collected from Loktak Lake gave less PC on minimal medium supplemented with 10 g/L fructose concentration at 37 °C. It was [49] reported poly (3-hydroxybutyrate-*co*-3-hydroxyhexanoate) P(3HB-*co*-3HHx) from palm olein and fructose using recombinant negative *Cupriavidus necator* Re2058/pCB113 strains. Fructose is naturally a homopolymer that is able to produce a P (3HB) when *Cupriavidus necator* Re2058/pCB113 strains grew on fructose (5 g/L fructose) as a sole carbon source). A 2.32 g/L CDW and 11% PHA/CDW homopolymer P (3HB) was produced from fructose, which is a higher CDW (g/L) and with less PC than the present study [49].

It has been observed that LPPI-18 is able to give a good PHA yield which is the closest yield to *B. cereus* SPV strain (38.0% ± 0.03%) [50]. It was also reported that *Cupriavidus* sp. KKU38 strains are capable to produce PHA when growing on cheap carbon sources like glucose, fructose, maltose, and xylose. Above all, glucose is the best carbon source for PHA production and has abilities to produce a 73.88% yield of PHA [8]. It is a higher PHA yield than our recent finding for newly screened *Bacillus* sp. LPPI-18 from Loktak Lake sediment suggesting that it could be due to the type of isolate used or other culture condition.

Fewer PCs were obtained from molasses when compared to glucose and fructose. Despite the fact that molasses are the potential substrate for PHA production, its level of PHA production is less than the same amount of glucose and fructose concentration which is probably due to the presence of sucrose concentration which is a double sugar broken by bacteria. Potential isolates of *Bacillus* sp. are able to produce PHA once grown on a minimal salt medium which is supplied with a carbon source. The estimated PHA contents are falling between 11.5 and 36.8% [51]. Different bacterial species are able to develop various amounts of PHA granules at 37 °C. For instance, the highest PHA contents were obtained at 24 h of fermentation for *Bacillus* sp. After 24 h of incubation, the PHA content was dropped which is inconsistent with our current results [51]. It was also reported a 42.10% PHA content for *Bacillus megaterium* BA-019 when grown on molasses [52] which is a higher yield than the current result.

Certain *Bacillus* species are able to produce PHA during fermentation. However, their DNA amplification for PHA gene did not occur when it was screened by using a primer. This might be a variation of gene type for PHA production. For instance, *B. megaterium* is able to produce PHA even though this isolate may not contain a gene of interest for PHA synthesis [51]. For the last 10 years, the polymers were produced from low-cost carbon source by using some potential industrially candidate pure cultures [53]. Molasses were used as a carbon source for PHA production when mixed with other organic acids. When sugar and organic acid are used, the conversion rate of PHA yield was not based on pH value. However, a slightly higher PHA yield is obtained when there are more acidic conditions. It has been reported that the highest yield of PHA was obtained from molasses which include about 0.22 g dry mass of PHA per 1 g of molasses. The PHA productivity was also 0.43 g/1 h in which it was in the range of reported PHA yield from other low-cost carbon sources from mixed cultures [53].

D-ribose can be used as a carbon source for microbial growth. Their role can be variously based on the type of microbial cells and culture condition. For instance, it was reported that new *Bacillus* sp. N-2 strains are also able to oxidize D-ribose when used as a carbon source. But the PHA production was not reported for this isolate [54]. In contrast to our recent finding, Diard et al. [55] employed D-ribose, and other carbon sources such as D-gluconate, D-galacturonate, itaconate, L-arabinose, L-tartrate, meso-tartrate, and tricarballylate for differentiation of *Pseudomonas* sp. GPo1 from *P. monteilii* and *P. putida* biotype A. However, PHA accumulating LPPI-18 isolate obtained from Loktak Lake is unable to utilize sucrose as a carbon source. Since the sucrose is a double sugar, it is hard for this isolate to break and be used as a carbon source. In agreement with the current study, it was [8] stated that some double sugars such as lactose, maltose, and sucrose were not utilized for *A. eutrophus* growth and PHA production.

All bacteria cannot grow on sucrose. For instance, it was recognized that *Ralstonia eutropha*, one of the most potential PHA-producing bacteria, is unable to grow on sucrose. However, when genetically engineered by expression of the *M. succiniciproducens sacC gene* encoding *b-fructofuranosidase*, *R. eutropha* is able to produce P(3HB) and P(3HB-co-LA) using sucrose as a carbon source (Park, *et.al*., 2015). Some bacterial spp. such as *Azotobbacter vinelandii*, *Alcaligens latus*, and *Hydrogenophage pseudoflaw*a have been identified as PHA-producing bacterial species [56, 57]. PHA-producing isolate has shown higher PHA producers. This may suggest that the LPPI-18 isolate is able to harbor genes and enzyme associated with respective enzyme for PHA polymerization. This bacterial isolate has been found to utilize carbon source such as glucose, ribose, and other cost-effective and cheap carbon source for other metabolic products.

PHA film and its pore can be seen with SEM when the film has a coralloid surface. The pores can be generated and observed within the PHA film (PHB), and the crystallization degree and/or rapid crystallization rate of these biodegradable plastics were occurring. The more the contents of PHA like PHBHHx which is considered to be films, the lesser the size of the protrusions and the pores of the film surfaces. A PHA polymer that is only made up of 3-hydroxybutyric acid (98.7%) is able to form films [22].

Its structure was typically polyester, one of ester functional groups. *Bacillus* sp.LPPI-18 has shown similar fingerprinting parts which matched with PHB standard, natural polyester (*sigma*). In agreement with this results, Oliveira et al. [58] obtained absorption band at 1725 and 1277cm^−1^ (Fig. 9) that corresponds to a stretch of C=O bond, which is a functional group. The same authors stated that a series of intense bands located at 1000–1300 cm^−1^ corresponds to the stretching of the C–O bond which is an ester group. A biopolymer sample was extracted from *Cupriavidus* sp. KKU showed an intense band at 1731.61cm^−1^ wave number that corresponds to a stretch of C=O functional group when it was characterized with FTIR [8].

The intense band at 1280 to 1050 cm^−1^ indicated the presence of C-O-C atoms, vibrations of the aliphatic ester group. Similarly, intense absorption band was obtained at 1280, 1227, and 1181 cm^−1^, spectra attributed to the vibrations of the aliphatic ester group (C-O-C) which is a related finding with our current results [59]. The collective structure and functional groups of this FTIR spectrum indicate the type of polyester. This polymer might be PHB, a carbon four polyester which is a class of PHA that is produced by certain *cyanobacteria* and heterotrophic bacteria [60].

## Conclusion

Generally, the following points are driven regarding the current study about biological degradable PHA production: A new potential bacterium species was screened from sediment. This isolate belonged to *Bacillus* species when it was characterized by using biochemical test and 16srRNA. Based on a combination of biochemical test and 16srRNA gene sequence, it was named as *Bacillus* sp. LPPI-18. It was able to produce PHA when this isolate was provided by 1% carbon source such as D-fructose, glucose, molasses, D-ribose, glycerol, and sucrose. In this study, more PHA content was obtained from glucose and less content was obtained from D-ribose. No PC was obtained from glycerol and sucrose for this isolate. It was also found that no PHA content was obtained from medium devoid of any carbon source. Variations (*P* ≤ 0.05) of PCs were observed among carbon source utilized for PHA productions. Following extraction, PHA films were prepared for these PHA-producing *Bacillus* species which is a brittle feature like PHB standard polymers. The PHA produced in this study was characterized by using FTIR, and it was matched with commercial PHB and hence identified as PHB.

## Supplementary Information

**Additional file 1: Table S1.** Sequence similarity for the recent PHA producing isolate and other closely related strain obtained from Eztaxon-e sever.

## Data Availability

All discussed data have been included into this manuscript.
