# Correction to: Polyhydroxyalkanoate recovery from newly screened *Bacillus* sp. LPPI-18 using various methods of extraction from Loktak Lake sediment sample

**DOI:** 10.1186/s43141-022-00418-0

**Published:** 2022-09-22

**Authors:** Seid Mohammed, Lopamudra Ray

**Affiliations:** 1 Department of Applied Biology, SoANS, Adama Science and Technology University, Oromia, Ethiopia; 2 School of Law, KIIT University, Bhubaneswar, Odisha 751024 India

**Correction: J Genet Eng Biotechnol 20, 115 (2022)**

**https://doi.org/10.1186/s43141-022-00392-7**

Following publication of the original article [1], the authors identified errors in Table 1; the third column header indicated ‘*Bacillus* sp. BNPI-92‘ while it should read ‘*Bacillus* sp. LPPI-18‘ and affiliation 3 should be removed and in addition Seid Mohammed is only affiliated with affiliation 1. The correct Table 1 is given below and the correct affiliations are given for both authors in this article.

**Table 1:** Biochemical test and enzyme assay

No	Type of assay	*Bacillus* sp. LPPI-18	*Bacillus cereus* ATCC 14579^T^ (Boone et al., 2001; de Vries et al., 2004; Ivanova et al., 2003)
1	Sample source	Soil sediment	Dairy
2	Morphology	Short rod	Rod shaped
3	Temperature for growth	25-37^o^C	10-45 ^o^C
4	Fresh plate culture	No smell	Mousy smell
5	2M NaCl	-	NP
6	Colony color	White	White to cream and sometimes Pinkish brown
7	1.5 M NaCl	-	NP
8	pH range	7-11	7
9	Gram staining	+	+
10	Endospore	+	+
11	Citrate test	-	+
12	TSI test (H_2_S production)	+	-
13	Bile salt	-	+
14	Methyl red test	+	+
15	Enzyme		
15.1	Amylase test	+	-
15.2	Catalase test	+	+
15.3	Pectinase test	-	+
15.5	Protease test	+	+
16	Gelatinase	-	+
17	D-fructose	+	-
18	D-glucose	+	-
19	D-ribose	+	+
20	Glycerol	-	+
21	PHA production on Glycerol	-	NP
22	PHA production on Crude oil waste	-	NP
23	PHA production on Rice Bran	-	NP
23	PHA production on Wheat bran	-	NP

*NP* not performed

The original article [1] has been corrected.
