# Supplementary material for: Polyhydroxyalkanoate recovery from newly screened Bacillus sp. LPPI-18 using various methods of extraction from Loktak Lake sediment sample
Source: J Genet Eng Biotechnol. 2022 Aug 6;20:115. doi: 10.1186/s43141-022-00392-7 (PMC9357249; doi:10.1186/s43141-022-00392-7)
Supplement: Supplementary file 1 — Additional file 1: Table S1. Sequence similarity for the recent PHA producing isolate and other closely related strain obtained from Eztaxon-e sever. [file 43141_2022_392_MOESM1_ESM.docx]

**Table-S_1_: Sequence similarity for the recent PHA producing isolate and other closely related strain obtained from Eztaxon-e sever**

| S.N. | Name related strains and the recent isolate | Strains | Accession | GC% | Sequence similarity with newly isolates recent strain (%) |
| --- | --- | --- | --- | --- | --- |
| 1 | *Enterobacter* sp. | LPPI-18 | ON678114 | 53.10 | recent isolate |
| 2 | *Bacillus cereus* | ATCC 14579^T^ | AE016877 | 53.53 | 99.01 |
| 3 | *Bacillus wiedmannii* | *FSL W8-0169* ^T^ | LOBC01000053 | 53.46 | 98.94 |
| 4 | *Bacillus paramycoides* | NH24A2^T^ | MAOI01000012 | 53.60 | 98.94 |
| 5 | *Bacillus megaterium* | TA-59 ^T^ | LC566119 | 53.58 | -- |
| 6 | *Bacillus paranthracis* | Mn5^T^ | MACE01000012 | 53.60 | 98.94 |
| 7 | *Bacillus megaterium* | Not known | FR715572 | 52.37 | -- |
| 8 | *Bacillus albus* | N35-10-2^T^ | MAOE01000087 | 53.46 | 98.94 |
| 9 | *Bacillus luti* | TD41^T^ | MACI01000041 | 53.46 | 98.94 |
| 10 | *Bacillus nitratireducens* | 4049^T^ | KJ812430 | 53.60 | 98.94 |
| 11 | *Bacillus proteolyticus* | TD42^T^ | MACH01000033 | 53.39 | 98.87 |
| 12 | *Bacillus tropicus* | N24^T^ | MACG01000025 | 53.53 | 98.87 |
| 13 | *Bacillus fungorum* | 17-SMS-01^T^ | MG601116 | 53.53 | 98.87 |
| 14 | *Bacillus anthracis* | Ames | AE016879 | 53.53 | 98.87 |
| 15 | *Bacillus pacificus* | EB422T | KJ812450 | 53.66 | 98.80 |
| 16 | *Bacillus toyonensis* | BCT-7112^T^ | CP006863 | 53.60 | 98.73 |
| 17 | *Bacillus mobilis* | 0711P9-1^T^ | MACF01000036 | 53.60 | 98.73 |
| 18 | *Bacillus thuringiensis gv. thuringiensis* | ATCC 10792^T^ | ACNF01000156 | 53.60 | 98.73 |
| 19 | *Bacillus clarus* | ATCC 21929^T^ | MH918154 | 53.60 | 98.66 |
| 20 | *Bacillus pseudomycoides* | DSM 12442^T^ | ACMX01000133 | 53.87 | 98.59 |
| 21 | *Bacillus mycoides* | DSM 2048^T^ | ACMU01000002 | 53.19 | 98.52 |
| 22 | *Bacillus bingmayongensis* | FJAT-13831^T^ | AKCS01000011 | 53.84 | 98.23 |
| 23 | *Bacillus manliponensis* | BL4-6^T^ | FJ416490 | 53.36 | 97.14 |
| 24 | *Micrococcus luteus* | DSM 20030^T^ | AJ536198.1 | 56.98 | -- |
